# MITOCHONDRIA AS REVERSIBLE REGULATORS OF AGING ASSOCIATED SKIN WRINKLES AND HAIR LOSS IN MICE

**DOI:** 10.1093/geroni/igz038.1461

**Published:** 2019-11-08

**Authors:** Keshav K Singh

**Affiliations:** University of Alabama at Birmingham, Birmingham, Alabama, United States

## Abstract

To evaluate the consequences of the decline in mtDNA content associated with aging we have created an inducible mouse model expressing, in the polymerase domain of POLG1, a dominant-negative mutation that induces depletion of mtDNA. We utilized this inducible mouse model to modulate mitochondrial function by depleting and repleting the mtDNA content. We demonstrate that, in mice, ubiquitous expression of dominant-negative mutant POLG1 leads to 1) reduction of mtDNA content in skin, 2) skin wrinkles, and 3) hair loss. By turning off the mutant POLG1 transgene expression in the whole animal, the skin and hair phenotypes revert to normal after repletion of mtDNA. Thus, we have developed whole-animal mtDNA depleter-repleter mice. These mice present evidence that mtDNA homeostasis is involved in skin aging phenotype and loss of hair and provide an unprecedented opportunity to create tissue-specific mitochondrial modulation to determine the role of the mitochondria in a particular tissue.

